# Longitudinal Influences on Maternal–Infant Bonding at 18 Months Postpartum: The Predictive Role of Perinatal and Postpartum Depression and Childbirth Trauma

**DOI:** 10.3390/jcm14103424

**Published:** 2025-05-14

**Authors:** Maria Vega-Sanz, Ana Berastegui, Alvaro Sanchez-Lopez

**Affiliations:** 1 University Institute of Family Studies, Pontifical Comillas University, 28108 Madrid, Spain; a.berastegui@comillas.edu; 2 Department of Personality, Evaluation and Clinical Psychology, Complutense University of Madrid, 28223 Madrid, Spain

**Keywords:** bonding, perinatal depression, posttraumatic stress, postpartum depression, longitudinal

## Abstract

**Background:** This study investigated the pathways through which various psychological problems occurring across the perinatal period influence mother–child bonding within the first 18 months postpartum, with a particular focus on the relationship between perinatal and postpartum depression and childbirth-related posttraumatic stress symptomatology. **Methods:** A multi-stage longitudinal design included three assessment points: recruitment and initial assessment in the third trimester of pregnancy (T1), a second assessment at 8 months postpartum (T2), and a final assessment at 18 months postpartum (T3). A total of N = 51 mothers completed all three waves (total follow-up period per participant: approximately 21 months). Sociodemographic data were collected, and all assessments were completed online. **Results:** Our findings revealed significant indirect effects, linking higher levels of perinatal depressive symptoms in the third trimester with mother–child bonding difficulties at 18 months postpartum. This association was mediated by both greater childbirth-related posttraumatic stress symptoms and elevated postpartum depressive symptoms at 8 months. **Conclusions:** These preliminary results highlight how complex perinatal factors at different stages (i.e., during pregnancy and early postpartum) influence mother–child bonding at 18 months postpartum. Understanding these pathways is essential in order to inform targeted interventions and to promote optimal maternal mental health and bonding outcomes.

## 1. Introduction

Mother–child bonding involves the mother’s mental representations of her baby and caregiving behaviors [1]. This bond begins with the intention to have a child and continues throughout infancy, reflecting the emotional, behavioral, cognitive, and neurobiological connections that the mother develops toward her baby over time [2].

A successful bond, established during the first 18 months postpartum, leads to positive interactions and an emotional connection from the mother to her baby [3]. However, some women face challenges in establishing this bond, which can disrupt the mother’s emotional responses to her baby during early post-birth interactions and throughout the first postpartum year [4]. The prevalence of these difficulties ranges from 3% to 24% [5], and they have significant impacts on the baby.

Indeed, the presence of such difficulties in bonding can produce multiple neurological alterations [6], cognitive impairments [7], difficulties in emotional adaptation [8], and problems of social development in children [9], as the establishment of a secure attachment bond is crucial for the child’s emotional regulation and social competence [10]. Furthermore, disruptions in early bonding can lead to persistent difficulties in emotional regulation, increased vulnerability to anxiety, depression, and interpersonal problems, and even an increased risk of developing personality disorders later in life [11]. These bonding difficulties are a risk factor for the later development of children’s psychopathology during childhood and adolescence, contributing to long-term challenges in emotional and psychological well-being [11]. These consequences and the relatively high occurrence of bonding difficulties highlight the need for a better understanding of the factors during the perinatal period that contribute to their development. Previous research has focused on factors contributing to bonding difficulties during the postnatal period. However, most of these studies have concentrated on the postpartum stage, with little exploration of earlier factors that may influence bonding. Additionally, while much of the research has focused on bonding quality during the first year, the literature is scarce on bonding quality beyond the first year of life. Furthermore, an integrative analysis of the main predictors and their relationships over time is still lacking.

To address this gap, the present study examines, within a longitudinal framework, a set of well-established factors across different perinatal stages. These factors have previously been studied in isolation, starting with key predictors identified in the postpartum period.

### 1.1. Postpartum Psychopathology and Bonding Difficulties: Childbirth-Related Posttraumatic Symptoms and Postpartum Depression Symptoms

The most immediate predictors of bonding difficulties emerge in the postpartum period, specifically during the first mother–child encounter at birth. Childbirth can sometimes be perceived as a traumatic event, leading to the development of Perinatal Posttraumatic Stress Symptoms (P-PTSSs) [12], which increase the risk of bonding difficulties [13]. P-PTSSs resemble those of general posttraumatic stress [14], but they are specifically related to the childbirth experience and affect both the mother and the baby [15,16,17]. Infants whose mothers experience P-PTSSs exhibit poorer cognitive development [8], reduced exploratory behaviors, increased avoidance of their mothers, more disorganized behavior, and a greater tendency to cry [18].

For the mother, the occurrence of P-PTSSs predicts the subsequent emergence of bonding difficulties, which, as mentioned, also affect the baby [13,15,16,17]. P-PTSSs impact bonding quality through mothers’ difficulty coping with caregiving tasks [19] and reduced perceptions of self-efficacy [20]. Additionally, some studies suggest that the baby may serve as a reminder of the traumatic birth event, triggering feelings of anger, resentment, and even rejection, further hindering the mother’s ability to bond with her baby [15,16,21]. These feelings also lead to fewer positive emotions toward the baby [22]. Importantly, these issues do not only occur in the early postpartum period. At 14 months postpartum, P-PTSSs continue to be associated with higher levels of bonding difficulties [16].

Another pathway through which P-PTSSs may contribute to bonding difficulties over time is through its relationship with postpartum depression symptoms (PoDSs). After childbirth, the experience of a traumatic or challenging birth can sometimes lead to negative feelings (e.g., sadness, incapacity, or hopelessness) associated with the development of PoDSs [23], which are a clear risk factor for bonding difficulties. Infants of mothers with PoDSs often exhibit poorer cognitive functioning, difficulties in emotion regulation, sleep and feeding problems, and behavioral issues [23]. These infants are also at a higher risk of developmental delays and clinical depression in adulthood [24].

Like P-PTSSs, PoDSs are another significant predictor of bonding difficulties during the postpartum period [5]. Recent studies have shown that PoDSs impact bonding by limiting the mother’s experience of positive emotions toward her baby, leading to emotional detachment and potential emotional neglect, with the mother engaging with the baby only to meet basic needs [5].

Overall, both P-PTSSs and PoDSs present separate pathways of influence on the establishment of mother–child bonding, but they can also occur together, enhancing their effects on the development of bonding difficulties at 18 months [22]. Previous studies indicate that these two pathologies have a very high comorbidity, meaning the distress associated with P-PTSSs is linked to higher levels of PoDSs [17,24]. This comorbidity could be due to a causal relationship between the two, where P-PTSSs act as a predictor and trigger for PoDSs [17,24]. Additionally, this comorbidity may arise from both conditions sharing common risk factors, particularly those before the postpartum period, such as the presence of perinatal depression symptoms (PeDSs) during pregnancy [21,23,25], which may contribute to bonding difficulties by exacerbating related symptoms that emerge postpartum.

### 1.2. Perinatal Depression as an Early Risk Factor for the Development of Bonding Difficulties

PeDSs can begin during pregnancy and extend into the postpartum period, evolving into PoDSs [23,26]. In addition to the symptoms of typical major depressive episodes, mothers with PeDSs may struggle with feelings of inadequacy in caring for their baby, leading to guilt and fear of harming the baby [23]. Similarly to P-PTSSs and PoDSs, PeDSs also impact child development in multiple ways. They can lead to low birth weight; an increased risk of preterm birth; the need for neonatal care; the development of difficult temperaments [27]; challenges in socio-emotional, cognitive (especially language), and motor development [28]; and even an altered response to physical contact in children up to two years of age [29].

Furthermore, PeDSs can act as a risk factor for the development of postpartum symptoms, which are related to the risk of bonding difficulties. Previous studies have shown that PeDSs increase the likelihood of perceiving childbirth as a traumatic event, triggering P-PTSSs at 18 months postpartum [21,23]. PeDSs during pregnancy are also considered the most important predictor of PoDSs [25,26,30], with approximately 50% of pregnant women with depression developing postpartum depression [31].

Despite the well-established associations between pregnancy-related risk factors and postpartum bonding difficulties, there are very few studies that have longitudinally examined how these variables interact over time [32,33]. Although initial findings are promising, an integrative analysis of the temporal dynamics among these interrelated factors is still lacking.

**Figure 1:**
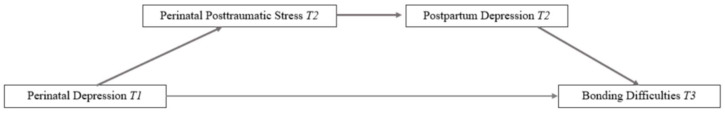
Path model proposed. T1: third trimester of pregnancy; T2: eight months postpartum; T3: eighteen months postpartum.

Therefore, this longitudinal study aims to identify the common and specific influences of perinatal and postpartum maternal mental health variables, as well as their mediating roles, on the development of bonding difficulties 18 months after childbirth. Specifically, we formulated and tested a mediational model (Figure 1) in which bonding difficulties at 18 months postpartum are indirectly predicted by psychological distress during the third trimester of pregnancy (PeDSs), through the sequential mediation of posttraumatic stress symptoms (P-PTSSs) and postpartum depressive symptoms (PoDSs) at 8 months postpartum. To examine this model, data were collected longitudinally at three time points: during the third trimester of gestation, at 8 months postpartum, and at 18 months postpartum.

## 2. Material and Methods

### 2.1. Design and Participants

This longitudinal study was conducted with three data collection phases using a snowball sampling technique to recruit the sample. Recruitment was conducted through the dissemination of the survey via social media platforms (such as Instagram and LinkedIn). The inclusion criteria were participants being women over 18 years old in the third trimester of pregnancy. The exclusion criteria applied were as follows: (1) being in a stage of pregnancy other than the third trimester, (2) being pregnant with more than one child. The first assessment (T1) comprised an initial sample of N = 594 pregnant women recruited from November 2020 to February 2021. The second assessment (T2) followed up on T1 participants eight months after giving birth, resulting in a total sample of N = 150, with data being collected between July 2021 and January 2022. The third assessment (T3) was performed eighteen months after giving birth (i.e., approximately 21 months since the initial recruitment), between May 2022 and December 2022, and had a final sample of completers of N = 51, which included those participants who had completed all the three assessments used in the path model tested in this study.

The sample attrition between phase T1 (third trimester of gestation) and phase T2 (eight months postpartum) was 75%; in the case of attrition between phase T2 and phase T3 (eighteen months postpartum), the sample attrition was 66%. Nonetheless, selective attrition tests were conducted on the sample, showing a low risk of attrition bias in the study. The results of these tests are reported in the Results Section.

The mean age of the final sample that completed the three assessments was 32.18 years old (SD = 4.46); 78.4% of the participants had completed university studies and 95% had a partner or were married at the time of the evaluation. Descriptive pregnancy, childbirth, and postpartum data are shown in Table 1. All the participating women signed an informed consent before participating in the study. The University Pontifical Comillas Ethics Committee approved the study protocol (2022/41).

### 2.2. Instruments

**Figure 2:**
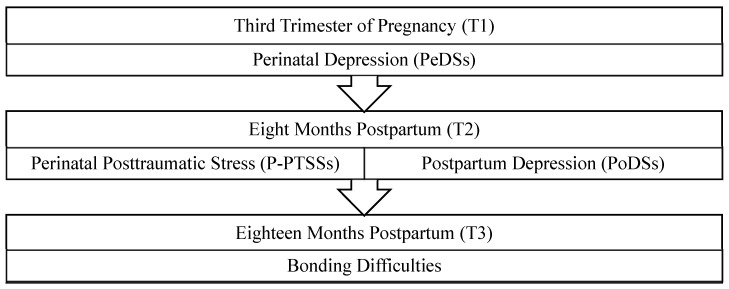
Phases and collected variables across evaluation process. T1: third trimester of pregnancy; T2: eight months postpartum; T3: eighteen months postpartum.

Data were collected through online evaluation surveys using commercial survey solutions, including questions about the previously reported sociodemographic data as well as standardized measures of the main psychological variables considered in the study. Figure 2 shows the measurement points of the study and the variables that were collected at each study phase that are relevant to the main analyses of this study.

The standardized questionnaires which assessed the relationship between PeDSs in T1, P-PTSSs, and PoDSs in T2, and mother–child bonding difficulties in T3 are presented below. The means, standard deviations, and alpha coefficients of all these dimensional psychological measurements are depicted in Table 2. All the variables showed good internal consistencies (see Table 2). For all analyses, the total sum of the items from each scale was used.

***Perinatal and Postpartum Depression.*** The Edinburgh Postnatal Depression Scale, EDPS [34], has been validated in the perinatal and postpartum stages. It is a 10-item scale, and its items are rated on a 4-point Likert scale. The cutoff score ≥ 13 indicates a high risk of depression during pregnancy, and the cutoff score ≥ 10 indicates a high risk of postpartum depression. It was applied twice, once to assess levels of PeDSs in T1 and another time to assess levels of PoDSs in T2. A total of 17% of the participants scored above the cutoff point at T1, and 36% did so at T2.

***Perinatal Posttraumatic Stress Symptoms.*** PCL-5, the PTSD Checklist for DMS-5, was used. The Posttraumatic Stress Disorder Symptom Checklist [35] comprises 20 items rated on a 5-point Likert scale. In this study, it was applied at T2, and it was specified in the instructions that mothers should complete this questionnaire based on their recent birth experience. A total of 9% of the women scored above the cutoff point on this questionnaire.

***Postpartum Bonding Difficulties.*** Finally, the Postpartum Bonding Questionnaire [36] was used to assess the presence of bonding difficulties, through items such as “I feel close to my baby” or “I wish the old days when I had no baby would come back”, at T3. It comprises 25 items that are rated on a 5-point Likert scale. This questionnaire provides a total score for bonding by summing up the scores of all its items.

### 2.3. Statistical Analysis

Statistical analyses were performed using IBM SPSS Statistics version 22 software. Initially, descriptive analyses of sociodemographic data and psychological measures of the participants were conducted. In addition, a preliminary test of selective attrition was conducted to ensure there were no differences between the completers and non-completers of the longitudinal assessments in the main variables under study. Subsequently, Pearson bivariate correlation analyses were carried out to test the association between mother–child bonding at eighteen months postpartum and the principal variables of the mediational model (i.e., predictors and mediators specified at each time point in Figure 1). We followed Cohen’s [37] criteria for interpreting correlation magnitudes.

As for our main analysis, we used a path model (i.e., Figure 1) to test the hypothesized mediational effects in the relationship between PeDSs and bonding difficulties at postpartum. In that model, PeDSs (T1) acted as the predictor variable, indirectly predicting bonding difficulties (T3) through the influence of P-PTSSs (T2) (i.e., Mediator 1), and PoDSs (T2) (i.e., Mediator 2). Bootstrapping (5000) was used to run the path analysis, following the recommendations of Preacher and Hayes [38]. The hypothesized mediation pathway (i.e., Figure 1) was tested by estimating indirect effects within the full path model and contrasting it with those considering alternative models controlling any of the mediators as covariates. This was performed using the package PROCESS for SPSS.

## 3. Results

### 3.1. Differences Between Completers and Non-Completers of Follow-Up Assessments

We conducted independent sample T-tests to evaluate the potential existence of selective attrition biases. The results indicated that there was no risk of biases in the estimation of the tested paths due to issues of attrition in the estimation of the variables. For psychological measures that were collected multiple times, no significant differences were found in the variables collected at T1 between completers and non-completers at T2 (PeDSs: t (592) = −0.335; *p* > 0.05; d = 0.15) nor between completers and non-completers at T3 (PeDSs: t (592) = 1.068; *p* > 0.05; d = 0.11). No differences were found in variables collected at T2 between completers and non-completers of T3 (PoDSs: t (148) = −0.60, d = 0.18; bonding difficulties: t (148) = −0.827, d = 0.10; both p’s > 0.05).

### 3.2. Bivariate Correlations Among Variables

Bivariate correlation analyses supported significant correlations between all the main variables of the model. The full set of results is shown in Table 3.

### 3.3. Mediational Model

**Figure 3:**
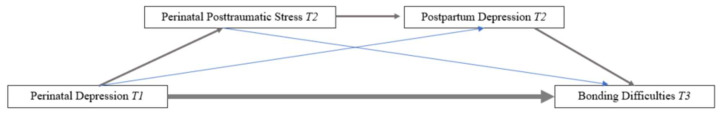
Mediation model between adaptation to pregnancy in third trimester of gestation and mother–child bonding at 18 months postpartum. T1: third trimester of pregnancy; T2: eight months postpartum; T3: eighteen months postpartum.

To test the mediational hypothesis, a model of indirect effects was carried out on the mediating role of the presence of P-PTSSs (T2; Mediator 1), and PoDSs (T2; Mediator 2) in the relationship between PeDSs (T1; Predictor) and mother–child bonding difficulties (T3; Outcome). Figure 3 fully specifies all the tested paths of relation among the proposed predictors and mediators.

Table 4 presents the full set of results of the indirect effect of the full formulated path model, as well as indirect effects for two alternative models in which one of the two postpartum symptomatology mediators is excluded from the path and controlled as a covariate. In the three supported models, PeDSs did not have direct effects on bonding difficulties at 18 months postpartum when controlling the proposed mediators as covariates.

Model 1 comprised the full path model hypothesized in the study. This full model did not show significant direct effects of PeDSs (T1) in the prediction of mother–child bonding difficulties (T3) without considering the modeled mediators. In contrast, in line with the hypothesized path, both the total and indirect effects of PeDSs (T1) on bonding difficulties (T3) through the full path of the mediators (P-PTSSs and PoDSs) were modestly significant, empirically supporting the proposed mediational model. In this model, PeDSs predicted higher levels of 0.02 standard deviations in bonding difficulties through their effect on the full path of the mediators, with higher PeDS levels at T1 predicting higher P-PTSS levels, which predicted higher PoDS levels at T2, the latter predicting higher bonding difficulties at T3.

In addition to Model 1, Models 2 and 3 were tested, eliminating one of the two mediators in each model, controlling them as covariates. Model 2 refers to a path not considering the mediating influence of PoDSs. In this model, the indirect effect was not significant. Model 3 refers to a path not considering the influence of P-PTSSs. In this model, the indirect effect was significant. Thus, PeDSs predicted higher levels of 0.01 standard deviations in bonding difficulties at 18 months through their effects on the modeled mediator (i.e., PoDSs at T2), with childbirth-related posttraumatic stress at T2 being controlled as a covariate.

## 4. Discussion

Previous research on postpartum mother–child bonding has primarily focused on the impact of maternal mental health in the immediate postpartum period or within the first year after childbirth. However, few studies have extended the analysis of these influences beyond this one-year boundary [32,33,39]. This is a limitation, as several studies show that difficulties in mother–child bonding beyond the first year are strong predictors of developmental challenges for children [7,9,40]. Therefore, in this study, we aimed to analyze various risk factors and their interactions to predict bonding difficulties at 18 months postpartum. Specifically, we sought to identify potential predictors during pregnancy and the postpartum period and test their specific relationships over time in order to predict bonding difficulties.

The path model tested in this study suggests that perinatal depression symptoms (PeDSs) during the third trimester of pregnancy are not only directly related to bonding difficulties 18 months after childbirth but may also contribute indirectly through their influence on different types of postpartum symptomatology. Our results indicate a potential indirect effect of higher PeDS levels (during the third trimester) on bonding quality at 18 months, mediated by the onset of postpartum childbirth-related posttraumatic stress symptoms (P-PTSSs) and depressive symptomatology. Specifically, P-PTSSs following a traumatic childbirth experience [12] could be a key mediator, leading to higher postpartum depressive symptoms (PoDSs), which ultimately contribute to the emergence of bonding difficulties [13,41]. These findings are in line with previous studies suggesting that PeDSs are a risk factor for both P-PTSSs [17,21,23] and clinical PoDSs [25,30]. In this way, depression during pregnancy could act as a risk factor for identifying childbirth as traumatic, leading to greater P-PTSS levels, which then trigger PoDSs and, in turn, increase bonding difficulties at 18 months (Model 1).

Additionally, Models 2 and 3 were tested, each considering a single postpartum symptom as a sole mediator. Model 3, which considered PoDSs at 8 months postpartum as the only mediating variable, presented significant indirect effects. These results suggest that P-PTSSs influence bonding quality at 18 months primarily through their effect on postpartum depression, with PoDSs emerging as the significant predictors of long-term bonding quality. This could be because postpartum depression significantly hinders the mother’s ability to emotionally engage with her baby, often limiting interactions to basic needs and leading to emotionally flat or neglectful caregiving [41]. In this sense, postpartum depression appears to be the most influential psychopathology on bonding at 18 months, while P-PTSSs affect bonding indirectly through their contribution to depressive symptoms.

However, it is important to note that these results are limited by the study’s sample size and should be viewed as a preliminary theoretical approach to the relationship between these variables, warranting further investigation. This study experienced a 75% sample attrition between phases T1 and T2, and 66% between phases T2 and T3. The sample loss throughout the different phases of the evaluation can be attributed to various factors. During initial recruitment, many mothers were first-time mothers and on pregnancy leave. However, by the final assessment, their children were 18 months old, and some mothers might have conceived another child, requiring additional maternity-related tasks. Additionally, some mothers might have returned to work, resulting in less available time. There may also have been a loss of motivation to participate due to the lack of compensation for participants or changes in the social context (e.g., no longer being in the COVID-19 pandemic), leading to reduced time or interest in participating in such studies.

Overall, these results highlight the need for further research with larger sample sizes and longitudinal designs to better understand the pathways of risk for the development of bonding difficulties. Such an approach would help refine our understanding of maternal and child health, providing insights into how early mental health issues influence the mother–child bond. These findings may also inform the development of training programs aimed at preventing bonding difficulties and raise awareness among healthcare professionals about prenatal, perinatal, and postpartum risk factors. Understanding how these factors unfold and relate over time can help professionals identify at-risk mothers, such as those with PeDSs. Early identification is crucial, as it can significantly impact mothers’ mental health postpartum and, ultimately, the mother–child relationship and the child’s future development. Our findings underscore the relevance of PeDSs as critical factors in preventing future postpartum symptoms and their effects on the mother–child bond. Similarly, the childbirth event itself is another very relevant phase due to the psychological processes that occur for both the mother and baby. These findings suggest the importance of implementing early interventions aimed at preventing bonding difficulties by addressing maternal symptomatology related to childbirth complications, which, as our results show, can be exacerbated by the presence of perinatal risk factors during the third trimester.

### Limitations and Strengths

Despite the importance of our findings, several limitations must be considered. Firstly, although there is an extensive literature on risk factors for the development of P-PTSSs [21,32] and PoDSs [23], due to sample size limitations and the need to control for numerous variables, not all of these factors were included in our analysis. While no selective attrition biases were found in the sample, significant sample loss occurred, particularly at T3 (i.e., about 21 months after initial assessment), which limits the interpretation of our results due to their potentially reduced statistical power. As a result, these findings should be taken with caution. However, these results pave the way for future research and the replication of this study with larger sample sizes. Their relevance lies in advancing new knowledge on risk pathways toward bonding difficulties, with both conceptual and practical implications. Secondly, concerning the recruitment method, participants recruited via social media and snowball sampling tend to share similar characteristics, which may affect the representativeness of the sample. This introduces potential selection bias that must be acknowledged. Additionally, there are other important limitations associated with the approach used. For instance, the evaluation was conducted online, which made it impossible to ensure that participants completed the questionnaires under optimal conditions. Factors such as distractions, fatigue, or lack of time may have influenced the quality of the responses provided. According to Kajdy et al. [40] and De Man et al. [41], this approach can also exclude women from low-income regions with lower socioeconomic or educational levels, further limiting the generalizability of the findings. Future studies should aim to expand predictive models, considering additional risk factors across different temporal stages, such as a history of depression or obstetric violence. In this study, symptoms were assessed in terms of dimensional levels of symptomatology at eight months postpartum. Future research should replicate our path model, incorporating assessments with additional temporal criteria and clinical relevance. The evaluation of P-PTSSs used a PTSD scale designed for the general population due to the lack of validated tools for the perinatal population. Furthermore, depressive symptoms during pregnancy and postpartum, as well as P-PTSSs, were assessed using screening tools, discussing the possible presence of high symptom levels rather than diagnosis with clinical disorders. This may impact on the results, highlighting the need for further research in this area.

Despite these limitations, several strengths of the study must be highlighted, such as the temporal analysis of factors involved in the development of P-PTSSs, their comorbidities with PoDSs, and their common and independent influences on bonding difficulties beyond the first year of children’s life. This represents a novel contribution to existing knowledge. While numerous studies [21,22] have examined both symptomologies across the postpartum period, there is limited research on their interplay in contributing to bonding difficulties, especially beyond the first years of children’s life. Finally, this research underscores the importance of studying the presence of different forms of psychopathology during the perinatal period and their impact on the childbirth and postpartum stages. To this end, we used an extensive longitudinal design, with assessments conducted at relevant stages, ensuring that our conclusions are reliable in terms of the specific mechanisms leading to bonding difficulties beyond the first year postpartum.

## 5. Conclusions

This study aimed to identify aspects of pregnancy and the postpartum period that impact bonding at 18 months of age and the mechanisms through which they contribute to mother–child bonding difficulties beyond the child’s first year. The results suggest that the relationship between difficulties with PeDSs and the subsequent occurrence of bonding difficulties (about two years after the initial difficulties) is fully mediated by P-PTSSs, in cases where the childbirth experience was traumatic, and PoDSs. Our results also indicate that PoDSs, triggered by PeDSs, independently impact bonding quality at 18 months to a greater extent (i.e., above and beyond the mediating role of P-PTSSs triggered after childbirth). Based on the longitudinal design, which evaluated various time points throughout pregnancy and the postpartum phase, these results provide valuable insights into perinatal psychology. They highlight the impact of maternal mental health during pregnancy and the postpartum period, extending beyond the first year of life, to better understand the crucial aspect of establishing a quality mother–child bond.

## Figures and Tables

**Table 1:** Descriptive pregnancy, childbirth, and postpartum data.

	Total Sample (N = 51)
Variable	%
Medical complications during pregnancy (T1)	
Yes	21.6
Type of delivery (T2)	
Natural	74.2
Cesarean section	0.3
Cesarean section with previous labor	16.9
Scheduled cesarean section	8.0
Home birth	0.6
Medical complications during childbirth (T2)	
Yes	28
Childbirth experience (T2)	
Satisfactory	58.2
Difficult but satisfactory	34.2
Traumatic	7.7
Medical complications after childbirth today (T3)	
Yes	12.0

T1: third trimester of pregnancy; T2: eight months postpartum; T3: eighteen months postpartum.

**Table 2:** Means, standard deviations, and alpha coefficients of dimensional psychological measurements.

*Instrument*	*Variables*	Phase	M (*SD*)	Alpha Coefficients (α)
EDPS	*Perinatal Depression Symptoms (PeDSs)*	T1	8.39 (*5.47*)	0.86
PCL-5	*Posttraumatic Stress Symptoms (P-PTSSs)*	T2	10.84 (*12.24*)	0.93
EDPS	*Postpartum Depression Symptoms (PoDSs)*	T2	18.08 (*9.24*)	0.88
PBQ	*Mother–child bonding*	T3	13.53 (*7.34*)	0.87

M = mean; SD: standard deviation; T1: third trimester of pregnancy; T2: eight months postpartum; T3: eighteen months postpartum.

**Table 3:** Bivariate correlations among the main psychological variables.

Measurer	1	2	3	4
1	1			
2	0.442 **	1		
3	0.513 *	0.728 **	1	
4	0.497 **	0.608 **	0.629 **	1

1: Perinatal depression symptoms (PeDSs, T1); 2: posttraumatic stress symptoms (P-PTSSs, T2); 3: postpartum depression symptoms (PoDSs, T2); 4: mother–child bonding difficulties (T3). T1: third trimester of pregnancy; T2: eight months postpartum; T3: eighteen months postpartum. * Correlations significant at *p* < 0.01. ** Correlations significant at *p* < 0.001.

**Table 4:** Analysis of total direct and indirect effects of mediational model.

	Independent Variable (VI)	Mediators (M)	Dependent Variable (VD)	Total Effect (e)	Direct Effect (e’)	Indirect Effect	95% CI (Indirect Effect)
Model 1	Perinatal Depression (PeDSs) (T1)	Posttraumatic Stress (P-PTSSs) (T2) Postpartum Depression (PeDSs) (T2)	Bonding (T3)	0.667 (SE = 0.166) (*p* = 0.000)	0.281 (SE = 0.166) (*p* = 0.961)	0.114 (SE = 0.068)	(0.020 to 0.314)
Model 2	Perinatal Depression (PeDSs) (T1)	Posttraumatic Stress (P-PTSSs) (T2)	Bonding (T3)	0.667 (SE = 0.166) (*p* = 0.000)	0.281 (SE = 0.166) (*p* = 0.961)	0.171 (SE = 0.115)	(−0.011 to 0.462)
Model 3	Perinatal Depression (PeDSs) (T1)	Postpartum Depression (PoDSs) (T2)	Bonding (T3)	0.667 (SE = 0.166) (*p* = 0.000)	0.281 (SE = 0.166) (*p* = 0.961)	0.098 (SE = 0.072)	(0.005 to 0.295)

T1: third trimester of pregnancy; T2: eight months postpartum; T3: eighteen months postpartum.

## Data Availability

Raw data are not publicly available in order to preserve individuals’ privacy under the European General Data Protection Regulation. The data used and/or analyzed during the current study is available from the corresponding author on reasonable request.
